# Identification of conserved hepatic transcriptomic responses to 17β-estradiol using high-throughput sequencing in brown trout

**DOI:** 10.1152/physiolgenomics.00123.2014

**Published:** 2015-06-16

**Authors:** Tamsyn M. Uren Webster, Janice A. Shears, Karen Moore, Eduarda M. Santos

**Affiliations:** Biosciences, College of Life & Environmental Sciences, University of Exeter, Exeter, United Kingdom

**Keywords:** RNA-Seq, Illumina, transcriptomics, sequencing, salmonid

## Abstract

Estrogenic chemicals are major contaminants of surface waters and can threaten the sustainability of natural fish populations. Characterization of the global molecular mechanisms of toxicity of environmental contaminants has been conducted primarily in model species rather than species with limited existing transcriptomic or genomic sequence information. We aimed to investigate the global mechanisms of toxicity of an endocrine disrupting chemical of environmental concern [17β-estradiol (E2)] using high-throughput RNA sequencing (RNA-Seq) in an environmentally relevant species, brown trout (*Salmo trutta*). We exposed mature males to measured concentrations of 1.94, 18.06, and 34.38 ng E2/l for 4 days and sequenced three individual liver samples per treatment using an Illumina HiSeq 2500 platform. Exposure to 34.4 ng E2/L resulted in 2,113 differentially regulated transcripts (FDR < 0.05). Functional analysis revealed upregulation of processes associated with vitellogenesis, including lipid metabolism, cellular proliferation, and ribosome biogenesis, together with a downregulation of carbohydrate metabolism. Using real-time quantitative PCR, we validated the expression of eight target genes and identified significant differences in the regulation of several known estrogen-responsive transcripts in fish exposed to the lower treatment concentrations (including *esr1* and *zp2.5*). We successfully used RNA-Seq to identify highly conserved responses to estrogen and also identified some estrogen-responsive transcripts that have been less well characterized, including *nots* and *tgm2l*. These results demonstrate the potential application of RNA-Seq as a valuable tool for assessing mechanistic effects of pollutants in ecologically relevant species for which little genomic information is available.

the major endogenous estrogen in vertebrates, 17β-estradiol (E2), is a significant contributor to the estrogenic contamination of surface waters, and E2 equivalent concentrations (EEQs) of up to 10 ng/l have been reported in rivers worldwide (10, 16, 26). In addition to input via wastewater treatment work effluents, E2 enters rivers via livestock and poultry waste and can cause pulses of contamination (50, 51). In water bodies, E2 can also act in conjunction with other natural and synthetic estrogenic chemicals (i.e., estrone; ethynylestradiol, phytoestrogens, alkylphenols and other industrial chemicals) to cause adverse effects in natural populations of fish. Reported effects of environmental estrogens include the induction of intersex in many species including roach (28) and gudgeon (58), decreased reproductive success in wild fish (21, 27), and population collapses (31), providing evidence for the risks that estrogens pose to the sustainability of wild fish populations.

The effects of E2, and other estrogenic contaminants, are mediated predominantly via genomic pathways through binding and activation of nuclear estrogen receptors, which are ligand-dependent transcription factors (48). Through this mechanism, estrogen exposure is associated with a highly conserved induction of a well-characterized suite of responsive genes. Of these, vitellogenin induction in male and juvenile fish has been the most widely described. In addition, induction of the transcripts encoding for estrogen receptor 1 (*esr1*) and zona pellucida proteins are also well-characterized responses to estrogen exposure (e.g., 18, 48, 55, 60). The transcription of these genes in the liver is known to be strongly associated with the stage of vitellogenesis in females and regulated via estrogen signaling (1). In addition to regulating the reproductive system, estrogens play a crucial role in a diverse range of other physiological processes including skeletal, muscular, cardiovascular, immune, and ion-regulatory systems, all of which are therefore potential targets for disruption following exposure to estrogenic contaminants in fish (19, 48).

Transcriptomic approaches have been employed to characterize both the normal endogenous effects of estrogen signaling in females, and the effects of exposure to a number of estrogenic chemicals in male and juvenile fish using microarrays (e.g., 4, 18, 30, 34) and high-throughput sequencing (RNA-SAGE) (60). These studies have reported extensive transcriptional changes, reflecting the diverse range of genes and processes regulated by estrogens, including a number of broadly conserved pathways. High-throughput RNA sequencing (RNA-Seq) has recently emerged as a robust, accurate, and reproducible tool for conducting transcriptomics (36, 43, 49), but, as yet, this approach has rarely been applied to ecotoxicology. A major advantage of this technique is that it can be used to conduct unbiased, global mechanistic analysis in any species of interest without a requirement for prior sequence information.

In this project, we employed RNA-Seq on an Illumina HiSeq 2500 platform to characterize the global hepatic transcriptomic responses of sexually mature male brown trout following exposure to E2, including at environmentally relevant concentration. Brown trout are an ecologically and economically important native European species, known to be sensitive to environmental stressors, but studies conducting mechanistic evaluations of its response to chemical toxicity are scarce. E2, originating from agricultural pollution, may be one of the environmentally relevant chemicals potentially affecting brown trout populations, which typically inhabit, and spawn in, smaller streams within farmland catchments. Additionally, by investigating the response to an estrogen, we aimed to discuss the suitability of RNA-Seq to identify a conserved mechanistic response and its role as a valuable and robust tool in ecotoxicology.

## MATERIALS AND METHODS

### 

#### Fish maintenance.

All experiments were conducted under approved protocols according to the UK Home Office regulations for use of animals in scientific procedures.

A mixed-sex population of brown trout (2 yr old) including mature and immature fish of both sexes were obtained from a local aquaculture facility (Hooke Springs Trout Farm, Dorset, UK) in late September, to correspond with the latter stages of reproductive maturation in this species, and maintained in 215-liter tanks to allow for acclimation to laboratory conditions for 3 wk prior to the start of the exposure, at the University of Exeter, UK. Each tank was aerated, supplied with 430 l/day dechlorinated tap water, and maintained at 12 ± 0.2°C, pH 7.5. Fish were kept under a 16:8 h light-dark cycle (with 30 min dawn/dusk transitional periods) and fed with pellet feed (8 mm; Biomar, Grangemouth, UK) at a rate of 2% body weight per day. To exclude sexually mature females, which would be naturally excreting estrogens that may have influenced the exposure, we measured plasma calcium concentrations in all fish prior to the start of the exposure. Concentration of plasma calcium is known to be a good indicator of vitellogenin and maturity status of female fish (40). Fish used in the exposure experiment included mature males and immature fish of both sexes.

#### Chemical exposures and sampling.

Chemical exposure was conducted via a flow through system for a period of 4 days. This exposure duration was chosen because short-term estrogen exposures are known to induce considerable and rapid transcriptional change (19) and to limit any possible confounding secondary effects of estrogen exposure (38). Four days can also be expected to simulate pulses of environmental estrogenic exposure associated with agricultural pollution (50, 51). Fish were exposed to three nominal concentrations, 2.5, 25, and 250 ng E2/l (17β-estradiol ≥98% purity, Sigma), or a dilution water control. The lowest concentration is in the range of EEQ concentrations reported in the environment (16, 26), while the higher concentrations were selected to facilitate mechanistic analysis.

Each treatment group consisted of one tank containing eight individual fish (mature males and sexually immature fish of both sexes), and the control treatment was run in duplicate. Water samples were collected from each tank on *day 3* of the exposure period and stored at −20°C prior to chemical analysis, using an Enzyme Immunoassay for Estradiol kit (Oxford Biomedical Research, Oxford, MI) according to the manufacturer's instructions. Samples were diluted or concentrated (using an appropriate ratio of ethyl acetate and exposure water) to fall within the range of assay detection (0.02–2 μg/l) and measured in duplicate. The cross-reactivity of this assay was 100% for E2 and ≤1.00% for testosterone and other sex steroids. The measured concentrations of E2 in the water were 1.94, 18.06, and 34.38 ng E2/l. The relatively low concentration of E2 measured in the 250 ng E2/l treatment group is likely due to its poor water solubility, given that we performed the exposure without the use of solvents to increase its environmental relevance. Concentrations of E2 in the concentrated stock solutions used to prepare the exposure concentrations were measured in parallel with exposure water samples and were 85, 77, and 43% of the nominal values for the 1.9, 18.1, and 34.4 ng/l treatments, respectively. This suggests that the poor recovery of E2 in the highest treatment concentration predominantly resulted from poor solubility in the stock solution. Throughout this paper, we refer to the measured concentrations of E2 to indicate the exposure concentrations.

Fish were humanely killed on *day 4* of the exposure period by a lethal dose of benzocaine (0.5 g/l, Sigma-Aldrich) followed by destruction of the brain by pithing with a blade, in accordance with UK Home Office regulations. Wet weight and fork length were recorded and the condition factor {k = [weight (g) × 100]/[fork length (cm)^3^]} was calculated for individual fish. Sex and maturity of all fish were confirmed by observation of the gonads, and gonadosomatic index (GSI) {[gonad weight (mg)/total weight (mg)] × 100 } was determined. Livers were dissected and weighed, and the hepatosomatic index (HSI) {[liver weight (mg)/total weight (mg)] × 100} was determined for individual fish. Portions of the liver were then snap-frozen in liquid nitrogen and stored at −80°C prior to RNA extraction. Statistical analysis of morphological parameters was conducted using SigmaStat (version 12.0). All morphometric data met assumptions of normality (verified by the Shapiro-Wilk test) and equal variance, and were analyzed by single-factor one-way analysis of variance (ANOVA).

#### RNA extraction, library preparation, and sequencing.

Transcript profiling was conducted in the livers of three sexually mature males per treatment group. RNA was extracted from livers with TRI reagent (Sigma-Aldrich) according to the manufacturer's instructions and then further purified and treated with DNase on RNeasy Mini extraction columns (Qiagen). The concentration, purity, and integrity of RNA were determined using a NanoDrop ND-1000 Spectrophotometer (NanoDrop Technologies) and an Agilent 2100 Bioanalyzer (Agilent Technologies). All RNA input to library construction was of high quality with 260/280 and 260/230 ratios >1.8 and RNA integrity number scores >8. External RNA Controls Consortium (ERCC) spike-in control mixes (Ambion) were added to all individual RNA samples, according to the manufacturer's instructions. cDNA libraries from all 15 samples were then prepared using the Illumina TruSeq RNA Sample Preparation kit, multiplexed with 24 samples per lane (together with samples from another project) and sequenced using an Illumina HiSeq 2500 to generate 100 bp paired-end reads, according to the manufacturer's instructions.

#### Transcriptome assembly and annotation.

To maximize sequence coverage depth and assemble an optimized male liver transcriptome for brown trout, we combined sequence reads from all samples from the current study with those from another project. Transcriptome assembly and annotation were conducted as described previously (57), and this transcriptome was then used as a basis for expression analysis in both projects. Contaminating Illumina adaptor sequences were removed, and the first 12 bp of all raw sequence reads were trimmed to remove 5′ bias caused by random hexamer priming using the FASTX-Toolkit (http://hannonlab.cshl.edu/fastx_toolkit, July 2013). 3′ sliding window quality trimming was performed (http://wiki.bioinformatics.ucdavis.edu/index.php/Trim.slidingWindow.pl, July 2013) and all reads where <90% bases had a Phred quality score >20 and those shorter than 15 bp were discarded. Digital normalization was performed to remove highly duplicated reads using the normalize-by-median.py script part of the khmer package described by Brown et al. (7), with the recommended k-mer value of 20 and a coverage threshold of 200. This process reduces the computer memory requirements of transcriptome assembly and also reduces the risk of potential sequencing error accumulation in abundant transcripts. All retained reads were then paired and separated into forward and reverse fastq files before de novo transcriptome assembly using Trinity (version r2013-02-25; Ref. 15, using the default parameters and specifying a minimum contig length of 200 bp). All transcripts were annotated using Blastx against Ensembl peptide databases (Release 71; April 2013) using an e-value cut off <1e^−15^ and assigned in the following preferential order: zebrafish (*Danio rerio*); human (*Homo sapiens*) and mouse (*Mus musculus*); then all other available fish species [stickleback (*Gasterosteus aculeatus*), medaka (*Oryzias latipes*), tilapia (*Oreochromis niloticus*), and cod (*Gadus morhua*)]. Additional annotation of previously unannotated differentially expressed transcripts was performed with Blast (e-value<1e^−15^) against NCBI RefSeq, nr, and nt databases.

#### Transcriptomic analysis.

Raw sequence reads from individual samples were mapped back against the assembled transcripts using Bowtie2 (version 2.1.0, Ref. 32), using the -k 1 parameter to report a single best hit for each read and limit ambiguous mapping to redundant transcripts. Raw count data for each transcript were extracted using idxstats in samtools (version 0.1.18, Ref. 35) and input into edgeR (45) for differential expression analysis. A criterion of at least one count in a minimum of three individual samples (corresponding to the number of individuals per treatment group) was imposed, and tagwise dispersion was applied with the recommended prior.df = 10. Comparisons were initially conducted between the two control groups to ensure that our analysis did not identify differential expression as a result of random variation between groups. Following this initial analysis, comparisons were conducted between the six individual fish from the combined control groups and three individuals from each of the other treatment groups. Transcripts were considered differentially expressed with an FDR < 0.05 (Benjamini-Hochberg correction). Hierarchical clustering was performed on all differentially expressed transcripts for all samples by an Euclidean distance metric, using the Pheatmap package for R. Functional analysis was then performed for differentially expressed genes from each treatment using the Database for Annotation, Visualization and Integrated Discovery (DAVID v6.7, Ref. 23), with the newly assembled brown trout male liver transcriptome as a background. KEGG pathways and Gene Ontology (GO) terms for biological processes, cellular components, and molecular functions were considered significantly overrepresented when *P* < 0.05. Canonical pathway and network analysis was conducted using Ingenuity Pathways Analysis (IPA; Ingenuity Systems, http://www.ingenuity.com) based on the list of differentially expressed transcripts.

The raw sequence data, and processed results from the expression analysis have been deposited in the National Center for Biotechnology Information's Gene Expression Omnibus (GEO) (http://www.ncbi.nlm.nih.gov/geo), and are available via the GEO series accession number GSE57490.

#### Real-time quantitative PCR analysis.

To validate the results of the differential expression analysis, real-time quantitative PCR (RT-QPCR) was used to quantify the expression of a selection of eight transcripts (*vtg1, nots, esr1, zp2.5, zp3a.2, crot, tat, tgm2l*) in the liver of all individual fish, including both sexually mature and immature males (*n* = 7, 5, 7, and 4 in the control and 1.9, 18.1, and 34.4 ng E2/l treatment groups, respectively). Transcript expression was also conducted for the remaining, immature female fish (*n* = 6, 2, and 2 in the control and 1.9, and 34 ng E2/l treatment groups, respectively), and analysis was conducted separately because of the very large sex differences observed for some transcripts (including *vtg1* and *nots*). Primers were designed using Beacon Designer 3.0 (Premier Biosoft International, Paulo Alto, CA), purchased from MWG-Biotech (Ebersburg, Germany) and optimized as previously described (56). The primer sequences, PCR product sizes, annealing temperatures, and PCR efficiencies for each optimized primer pair are shown in Table 1. cDNA was synthesized from 2 μg of total RNA treated with RQ1 DNase (Promega, Southampton, UK) using random hexamers (MWG-Biotech) and M-MLV reverse transcriptase (Promega), according to the manufacturer's instructions. RT-QPCR was performed using 1:2 diluted cDNA in duplicate, using SYBR green chemistry, with an iCycler iQ Real-time Detection System (Bio-Rad Laboratories, Hercules, CA), including a negative control run in duplicate on each plate to verify the absence of cDNA contamination. Efficiency-corrected relative expression levels for each transcript were determined by normalizing to a control transcript, *vapa*, which was selected based on its highly consistent expression across all individuals in the RNA-Seq dataset.

**Table 1.** Target genes, primer sequences, and assay details for RT-QPCR analysis

Target Gene	Symbol	Forward Primer (5′-3′)	Reverse Primer (5′-3′)	Product Size, bp	Ta, °C	PCR Efficiency, %
Vesicle-associated membrane protein-associated protein A	*vapa*	CACTGAACATTCCAACTC	TGAGCATTGATAACAGGT	118	59.5	95.5
Estrogen receptor 1	*esr1*	GCAGAACACTTCACAGCATT	ATCCACATAACAGCGACAGA	126	59.5	101.9
Carnitine O-octanoyltransferase	*crot*	GCTGGTAATGTGGTGTTG	ATGGTATCCTTGGTGACTC	83	53.5	105.0
Nothepsin	*nots*	ATGATGACAGGAGGTGAA	AGGAAGGAAAGAAGGAAGA	86	58.0	114.0
Tyrosine aminotransferase	*tat*	AGCATCGTAATCCTAGCAAGA	TCAAGCACCAGCACAGAT	83	56.0	95.7
Transglutaminase 2 like	*tgm2l*	CTGCCACCTAAACACAAA	ATCCAACACCTTCACAAC	75	56.0	99.9
Vitellogenin 1	*vtg1*	AACTTGATTGGAATTGAG	TAATACCTACTTGCTGAA	132	55.0	111.5
Zona pellucida glycoprotein 2.5	*zp2.5*	ATCAATAACCACAGCCACAATG	ACCAGGGACAGCCAATATG	75	55.0	101.2
Zona pellucida glycoprotein 3a.2	*zp3a.2*	AACTACACTCCACTTCATC	CACATCTCCTTCATCTTCA	86	54.5	112.6

RT-QPCR, real-time quantitative PCR; Ta, annealing temperature.

Statistical analyses of RT-QPCR data were conducted with SigmaStat (version 12.0). Transcript expression data that did not meet normally distributed criteria were log-transformed before statistical analysis. All data were analyzed by single-factor one-way ANOVA, followed by the Holm-Sidak post hoc test. Data were considered to be significant when *P* < 0.05.

## RESULTS

### 

#### Morphological parameters.

Visual examination of the gonads revealed there were 17 mature males, defined by the presence of large white testis and milt, across all treatment groups (*n* = 3–7 fish per treatment). The remaining fish were sexually immature males and females. The mean mass and length of all mature males were 472.3 ± 9.1 g and 34.3 ± 0.2 cm, of immature males were 430.5 ± 44.5 g and 32.6 ± 0.9cm, and of females were 420.0 ± 15.9 g and 33.5 ± 0.5 cm. The mean condition factors, HSI, and GSI were, respectively, 1.16 ± 0.01, 1.11 ± 0.03, and 3.95 ± 0.32 for mature males, 1.24 ± 0.12, 0.85 ± 0.04, and 0.06 ± 0.01 for immature males, and 1.11 ± 0.02, 1.00 ± 0.03, and 0.31 ± 0.02 for immature females, and there were no significant differences for these parameters between treatment groups. Additionally, we observed no alteration of general health or behavior during the exposure period.

#### Sequencing and transcriptome assembly.

In total, we sequenced 225.3 million paired 100 bp reads from male brown trout liver samples, and 208.1 million (92.4%) of these were retained after processing and quality filtering. As described in Uren Webster et al. (57), highly duplicated reads were then removed by digital normalization, and 46.73 million paired reads were retained for input into the de novo transcriptome assembly. The final transcriptome assembly consisted of 172,688 transcripts (107,095 loci) with a mean length of 767.5 bp and an N50 of 1,292 bp. Of these, 62,236 transcripts were annotated using Blastx (e-value < 1e^−15^) against Ensembl peptide databases, and these included representation of 16,121 unique zebrafish transcripts.

#### Transcript expression analysis.

**Fig. 1.**
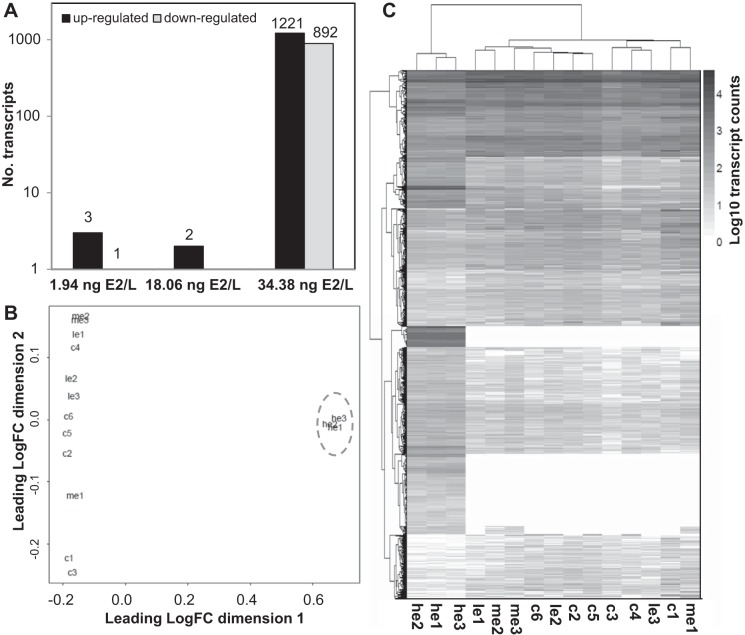
Differentially expressed transcripts following exposure to 17β-estradiol (E2) in the liver of mature male brown trout. Multiple transcripts are included for each gene annotation, which potentially reflect the presence of different isoforms as well as redundant fragments within the list of differentially expressed transcripts. *A*: number of upregulated and downregulated transcripts in each treatment group calculated with EdgeR [false discovery rate (FDR) <0.05]. *B*: multidimensional scaling plot illustrating the very significant effect of exposure to 34.4 ng E2/l on the hepatic transcriptome of male brown trout (presented within the blue circle, for visualization purposes) compared with all other groups, based on the expression of all differentially regulated transcripts. Individual fish are represented by the following codes: c1, c2, c3 c4, c5, and c6 represent the control individuals; le1, le2, and le3 represent individuals exposed to 1.9 ng E2/l; me1, me2, and me3 represent individuals exposed to 18.1 ng E2/l; he1, he2, and he3 represent individuals exposed to 34.4 ng E2/l. *C*: heat map illustrating the relative expression level of all differentially regulated transcripts in all individual samples (individuals are represented by the same codes as in *B*). Data presented are log10-transformed read counts per transcript. The hierarchical clustering to generate gene and condition trees was conducted using a Euclidean distance metric in the R pheatmap package.

A total of 137.6 million raw reads were obtained from the libraries generated from liver samples of E2-exposed and control male fish, averaging 9.2 million reads per individual sample, and 83.1% of these were remapped against the transcriptome assembly. Differential expression analysis between the control groups revealed only three differentially regulated transcripts. Comparisons between each E2 treatment group and the combined control group were conducted and resulted in only four (*bcl6a, spns1*, NM_001124310, uncharacterized transcript) and two (NM_001124310, uncharacterized transcript) differentially expressed transcripts for fish exposed to 1.9 and 18.1 ng E2/l, respectively. This may correspond to changes associated with tank effects, given that similar numbers of differentially expressed transcripts were found between the two control tanks, and no treatment associated trends were observed for any of these transcripts. Exposure to 34.4 ng E2/l, however, resulted in 2,113 differentially expressed transcripts (Fig. 1*A*, Supplemental Table S1), including 808 unique annotations.^1^ Multidimensional scaling plots and Euclidean cluster analysis based on all differentially regulated transcripts show that all three individual fish exposed to 34.4 ng E2/l have a very similar and consistent expression profile, clearly distinct from all other fish, whereas the control fish and those exposed to the lower concentrations of E2 cluster together (Fig. 1, *B* and *C*).

A list of the 20 most up- and downregulated transcripts following exposure to 34.4 ng E2/l is shown in Table 2. The greatest fold-changes in expression were associated with upregulated transcripts and were dominated by well-characterized estrogen-responsive genes, including a number of vitellogenin transcripts (*vtg1, vtg1l, vtg2, vtg3, vtg6, vtg7*), of which *vtg1* was the most highly expressed. Additionally, a transcript encoding nothepsin (*nots*) was similarly strongly induced in fish exposed to 34.4 ng E2/l. Transcripts encoding zona pellucida proteins (*zp2.2, zp2.5, zp3a.1, zp3a.2*) were also upregulated (up to 70- to 230-fold) and *esr1* was upregulated by up to 27-fold. There was a trend toward upregulation of *esr1, zp3a.2*, and *zp2.5* (2- to 4-fold) in the lower treatment groups compared with the control, but these results were not statistically significant, likely due to the low number of replicates in the RNA-Seq dataset.

**Table 2.** List of the 20 most upregulated and downregulated transcripts in fish exposed to 34.4 ng E2/l

Upregulated				Downregulated			
Symbol	Name^a^	Fold Change^b^	FDR	Symbol	Name^a^	Fold Change^b^	FDR
*vtg1*	vitellogenin 1	↑ >5438	4.6E-119	*tat*	tyrosine aminotransferase	**↓** 4.6	8.9E-9
*nots*	nothepsin	↑ >4475	5.4E-107	*tgm2l*	transglutaminase 2, like	**↓** 186.1	3.3E-8
*vtg6*	vitellogenin 6	↑ >2000	1.3E-102	*cbln8*	cerebellin 8	**↓** 5.7	2.6E-7
*vtg2*	vitellogenin 2	↑ >1100	7.3E-92	*hsd3b7*	hydroxy-delta-5-steroid dehydrogenase, 3beta- and steroid delta-isomerase	**↓** 5.4	2.8E-7
si:dkey-4c23.3	vitellogenin 1-1	↑ >220	9.6E-59	*errfi1*	ERBB receptor feedback inhibitor 1	**↓** 6.3	5.5E-7
*vtg3*	vitellogenin 3	↑ >825	1.4E-56	*igfbp1a*	insulin-like growth factor binding protein 1a	**↓** 50.7	6.2E-7
*zp3a.2*	zona pellucida 3a.2	↑ 149	1.6E-53	*slc3a2a*	solute carrier family 3, member 2a	**↓** 10.4	6.8E-7
si:dkey-179j5.2	family with sequence similarity 20, member C	↑ >185	5.0E-52	*faxdc2*	chromosome 5 open reading frame 4	**↓** 4.8	1.8E-6
*zp2.5*	zona pellucida 2.5	↑ 77.6	1.1E-42	*pnp5a*	purine nucleoside phosphorylase 5a	**↓** 27.1	2.3E-6
*zp3a.1*	zona pellucida 3a.1	↑ 161.5	1.1E-42	*epha8*	eph receptor A8	**↓** >21	7.5E-6
*vtg7*	vitellogenin 7	↑ >107	8.3E-42	*pfkfb1*	6-phosphofructo-2-kinase/fructose-2,6-biphosphatase 1	**↓** 30.1	8.1E-6
*crot*	carnitine o-octanoyltransferase	↑ 54.4	4.9E-40	*pptc7a*	PTC7 protein phosphatase homolog a	**↓** 6.3	9.2E-6
*esr1*	estrogen receptor 1	↑ 25.7	1.6E-37	si:dkey-238o13.4	si:dkey-238o13.4	**↓** 4.5	1.8E-5
*zp2.2*	zona pellucida 2.2	↑ 160.7	5.1E-31	*st3gal3b*	ST3 beta-galactoside alpha-2,3-sialyltransferase 3b	**↓** 3.8	2.4E-5
*aqp12*	aquaporin 12	↑ 28.6	8.4E-31	*ret*	ret proto-oncogene receptor tyrosine kinase	**↓** 8.5	4.3E-5
*lrrc58b*	leucine rich repeat containing 58b	↑ 20.6	1.0E-30	*ntng2a*	netrin g2a	**↓** 4.9	4.9E-5
*igfbp5a*	insulin-like growth factor binding protein 5a	↑ >49	2.6E-30	*ulk1a*	unc-51-like kinase 1a	**↓** 4.7	4.9E-5
*rdh10a*	retinol dehydrogenase 10a	↑ >108	8.8E-28	*grb7*	growth factor receptor-bound protein 7	**↓** 26.3	1.2E-4
*slc7a11*	solute carrier family 7, member 11	↑ >51	8.9E-27	*cldn11a*	claudin 11a	**↓** 6.5	1.3E-4
*lpgat1*	lysophosphatidylglycerol acyltransferase 1	↑ 26.0	3.0E-25	*slc25a29*	solute carrier family 25, member 29	**↓** 4.8	1.4E-4

^a^ Where there were multiple differentially regulated transcripts assigned the same annotation, only the most significantly regulated transcript is included in this list.

^b^ For transcripts where no read counts were detected in any of the individuals in one of the groups, a nominal value of 1 count was given to each individual in that group to calculate a fold change value, for visualization purposes, in this table. E2, 17β-estradiol; FDR, false discovery rate.

Analysis of ERCC spike-in control data was conducted to determine the accuracy and dynamic range of the transcript expression measurements in this study. For all individual samples, there was a strong correlation between the calculated FPKM (fragments per kilobase of transcript per million mapped reads) values and the expected concentration of control transcripts (mean R^2^ = 0.902 ± 0.005). The dynamic range was calculated for all samples individually, using the control transcripts that were detected in a minimum of three libraries as the lower cut-off limit. The mean dynamic range in expression level for all 15 libraries was 26,753 FPKM. There was also a good correlation between the calculated and expected changes in transcript expression level between samples spiked with ERCC *mix 1* and *mix 2* (R^2^ = 0.58). Together, these results provide strong technical validation for the quantitative expression profiling conducted in this study.

#### Functional analysis.

**Fig. 2.**
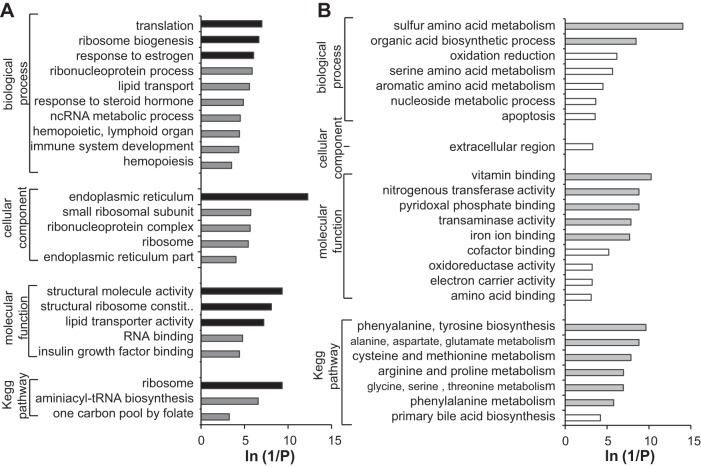
Overrepresented Gene Ontology (GO) terms and KEGG pathways (*P* < 0.05) in the list of upregulated (*A*) and downregulated transcripts (*B*) in fish exposed to 34.4 ng/l E2. Values presented represent the *P* value associated with overrepresentation. Darker shaded bars indicate GO terms where the adjusted *P* value was < 0.05 (Benjamini-Hochberg correction). Analysis was conducted using the Database for Annotation, Visualization and Integrated Discovery (DAVID) (Huang et al. 2008) v6.7, using our brown trout liver transcriptome as a background and Reduce and Visualize Gene Ontology (Revigo)(53) to condense redundant terms.

Enriched GO terms and KEGG pathways among up- and downregulated transcripts following exposure to 34.4 ng E2/l are illustrated in Fig. 2. GO terms including translation, ribosome, lipid metabolic processes, and growth factor binding were overrepresented in the list of upregulated transcripts. Regulated transcripts within these GOs include RNA polymerases (*polr1a, polr3a*) for transcription; translation initiation factors (*eif1ad, eif3s10, eif4a2*) for translation; and ribosomal components and binding proteins (*rpl5a, rpl12, rpl15, rpl36a, rpl39, rplp0, rpp21, rps2, rps9, rps23, rpsa, rrbp1a*) for ribosome. Within lipid metabolism, differentially regulated transcripts included apolipoproteins (*apob, apobb, apof, apoc2*), lipoprotein receptor (*lrpap1*), glycolipid transfer proteins (*gltpd2*), and transcripts involved in PPAR signaling (*ppardb, acoxl*). In addition, insulin-like growth factor (IGF) signaling was also affected and transcripts encoding IGF binding proteins (IGFBPs) were upregulated in some cases (*igfbp5a, igfbp2a, igfbp2b*) and downregulated in others (*igfbp1a, igfbp1b*).

For downregulated transcripts, the most overrepresented GO terms related to amino acid metabolism and biosynthesis and associated processes including organic acid biosynthesis, transaminase activity, and pyridoxal phosphate binding. Of note, a number of processes involved in cysteine and methionine metabolic pathways were enriched, whereby differentially regulated transcripts included betaine-homocysteine methyltransferase (*bhmt*), S-adenosylmethionine synthase (*sash1*), methionine adenosyltransferase (*mat2aa*), and cysteine dioxygenase (*cdo1*). Apoptosis and programmed cell death were also overrepresented in the list of downregulated transcripts.

**Fig. 3.**
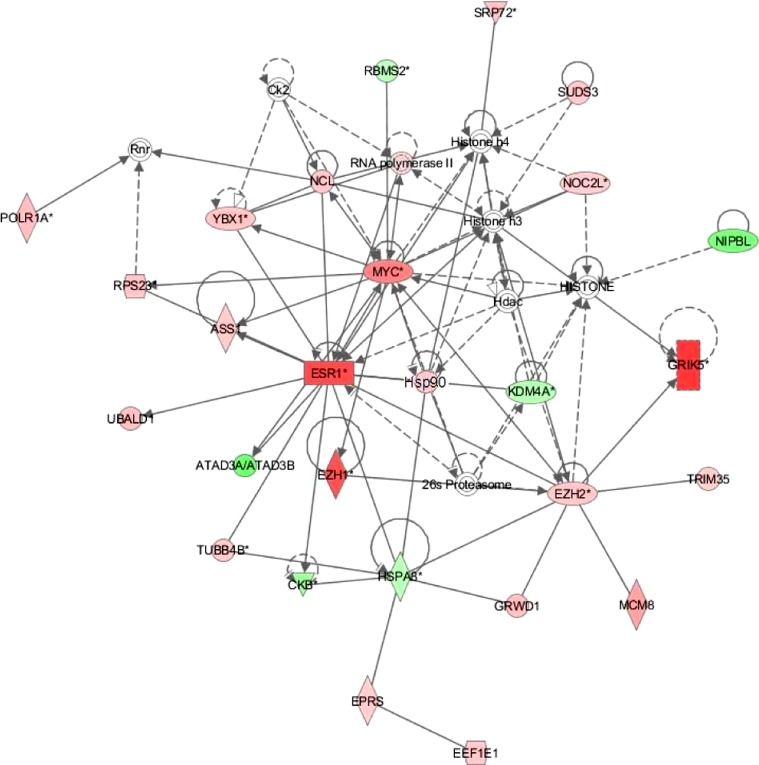
Enriched gene network constructed using differentially expressed transcripts (FDR <0.05) following exposure to 34.4 ng E2/l. This was the highest scoring enriched network generated by Ingenuity Pathway Analysis (IPA) on default settings. Associated functions of this network include amino acid metabolism, cell death and survival, endocrine system development, and small molecule biochemistry. Nodes represent genes and edges represent gene relationships. The intensity of node shading represents degree of upregulation (red) or downregulation (green), while uncolored nodes represent genes that were not identified as being differentially expressed in our experiment but were included in this network based on evidence stored in IPA databases. Node shapes denote enzymes, phosphatases, kinases, peptidases, G protein-coupled receptor, transmembrane receptor, cytokines, growth factor, ion channel, transporter, translation factor, nuclear receptor, transcription factor, and other. EEF1E1, eukaryotic translation elongation factor 1 epsilon 1; EPRS, glutamyl-prolyl-tRNA synthetase; HSPA8, heat shock 70 kDa protein 8; GRWD1, glutamate-rich WD repeat containing 1; CKB, creatine kinase, brain; TUB84B, tubulin alpha 1-like protein; MCM8, minichromosome maintenance complex component 8; EZH2, enhancer of zeste homolog 2; TRIM35, tripartite motif containing 35; EZH1, enhancer of zeste homolog 1; ATAD3A/ATAS3B, ATPase family, AAA domain containing 3A/3B; GRIK5, glutamate receptor, ionotropic, kainate 5; KDM4A, lysine (K)-specific demethylase 4A; Hsp90, 90 kDa heat shock protein; ESR1, estrogen receptor 1; UBALD1, UBA-like domain containing 1; NIPBL, Nipped-B homolog; MYC, v-myc avian myelocytomatosis viral oncogene homolog; ASS1, argininosuccinate synthase 1; RPS23, ribosomal protein S23; NOC2L, nucleolar complex associated 2 homolog; YBX1, Y box binding protein 1; NCL, nucleolin; POLR1A, polymerase (RNA) I polypeptide A, 194 kDa; SUDS3, suppressor of defective silencing 3 homolog; RBMS2, RNA binding motif, single stranded interacting protein 2; SRP72, signal recognition particle 72 kDa.

IPA identified a gene network involved in the response to E2 with functions relating to amino acid metabolism, cell death and survival, endocrine system development, and small molecule biochemistry, and with *esr1* and the myelocytomatosis oncogene (*myc*) as central nodes (Fig. 3). In particular, a number of genes and processes in this network can be associated with transcription and translation including histones, RNA polymerase, and several translation elongation factors.

#### RT-QPCR validation.

**Fig. 4.**
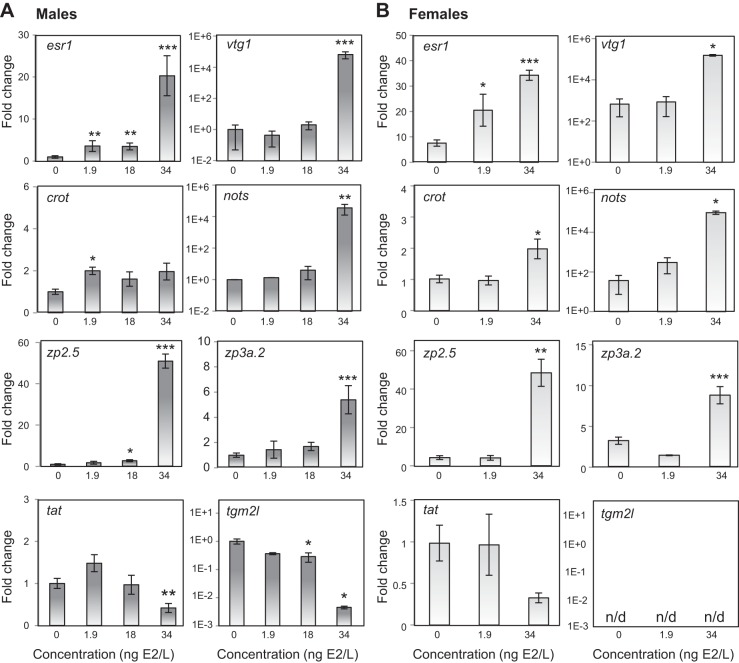
Transcript profile analysis for a selection of target genes in all males (*A*) and females (*B*), conducted by RT-QPCR. Data are presented as mean fold-change relative to expression in the control group. Relative expression was calculated as ratio of the expression for each target gene/expression for *vapa* mRNA. Expression of *tgm2l* was quantified only in mature males and was below the detection limit of the RT-QPCR assay for both immature males and females (n/d, nondetectable). Data were collected from 3–7 males and 2–6 females per treatment group. Individuals for which the expression was below the detection limit of the assay were excluded from the analysis. Asterisks represent significant differences between each treatment group and the control group (*P < 0.05, **P < 0.01, ***P < 0.001).

RT-QPCR analysis, performed for eight transcripts using all of the male fish, fully validated the results of the RNA-Seq expression analysis (Fig. 4*A*). Five upregulated transcripts (*vtg1, nots, esr1, zp2.5, zp3a.2*) and two downregulated transcripts (*tat, tgm2l*) were confirmed as being significantly differentially expressed in fish exposed to 34.4 ng/l E2 compared with those in the control group. Furthermore, the expression of *esr1* was also significantly increased in both the 1.9 and 18.1 ng/l groups, while *zp2.5* and *tgm2l* were significantly up- and downregulated, respectively, following exposure to 18.1 ng/l, confirming the trends identified in the RNA-Seq dataset. For *crot*, there was a significant increase in expression of this transcript in male fish exposed to 1.9 ng/l E2 and increasing, but nonsignificant, trends in expression in the two higher treatment groups. RT-QPCR analysis was also performed on the same transcripts in the immature female fish and identified very similar patterns of expression including significant upregulation of *vtg1,nots, zp2.5, zp3a.2, crot* in the highest treatment group, and significant upregulation of *esr1* in fish exposed to both 1.9 and 34.4 ng/l E2 (females were not present in the group of fish exposed to 18.1 ng/l; Fig. 4*B*). *tgm2l* expression was detected by RT-QPCR only in sexually mature males (*n* = 15 across all groups) and not in any immature males (*n* = 6) or females (*n* = 10).

## DISCUSSION

Despite the ecological and economic importance of brown trout, relatively little is known about the responses of this species to key stressors affecting its freshwater habitat, including endocrine disrupting chemicals. Here, we have conducted global transcriptional profiling using RNA-Seq in the liver of sexually mature males exposed to E2 and identified very significant transcriptional changes at the highest concentration tested (34.4 ng E2/l) that were very consistent between the three individual fish in this group. In contrast, concentrations of up to 18.1 ng E2/l did not induce significant changes following 4 days of exposure. RT-QPCR analysis, using a greater number of individual fish per treatment, fully validated the results of the RNA-Seq analysis for eight target genes (including both up- and downregulated transcripts) and also identified significant differences in expression of several of these in the 1.9 ng E2/l group (*esr1* and *crot*) and 18.1 ng E2/L group (*esr1, zp2.5*, and *tgm2l*). Importantly, this demonstrates that short-term exposures to low, environmentally relevant concentrations of E2 induce significant changes in the expression of some of the most sensitive estrogen-responsive genes in brown trout. Although 34.4 ng/l is far higher than EEQ concentrations regularly reported in surface waters, it is within a range reported to occur in treated sewage effluent (16, 26) and may be associated with short-term peaks of E2 contamination in streams inhabited by this species that occur as a result of agricultural pollution (51). Therefore, the extent of transcriptional change found in this study after a 4-day exposure to 34.4 ng E2/l may also be of interest for evaluating the potential impact of estrogens on populations of brown trout in the most contaminated environments.

### 

#### Conserved estrogen-responsive transcripts.

Transcripts encoding six vitellogenin isoforms were very strongly induced in males exposed to 34.4 ng E2/l but were not detected by RNA-Seq in fish exposed to 18.1 ng/l and below. RT-QPCR quantified very low levels of *vtg1* expression in males in the control group and confirmed a very significant upregulation in males exposed to 34.4 ng E2/l (by 66,000-fold), but no significant difference in expression in the two lower treatment groups. These results are similar to those reported in previous transcriptomic studies where *vtg* transcripts are generally the most strongly upregulated following estrogen exposure (e.g., 34, 60). The threshold for induction of vitellogenin transcript expression in mature males in this study was slightly higher than previously reported values for transcript and protein induction in salmonids. In juvenile rainbow trout exposed to E2 for 14 days, the median effective treatment concentration for plasma Vtg protein induction was in the range of 19–26 ng/l (55), while the lowest effective concentration for both plasma Vtg protein and hepatic *vtg1* transcript induction was found to be 14 ng/l (54). In juvenile brown trout, the median EC50 for plasma Vtg protein induction following 7-day E2 exposure was 15 ng/l (5). Here, this relatively higher threshold level for vitellogenin induction compared with previous reports for E2 exposure may reflect the shorter exposure period in this study of only four days and/or differences in maturity status.

Transcripts encoding four zona pellucida proteins and *esr1* were also among the most upregulated transcripts in fish exposed to 34.4 ng E2/l, similar to previous reports showing strong upregulation in vitellogenic females and induction by E2 in males (18, 34, 60). There were also nonsignificant trends toward upregulation of *zp*s and *esr1* in the lower treatment groups (by 2- to 4-fold), and RT-QPCR confirmed significant upregulation of *esr1* (in males and females) from 1.9 ng E2/l, and of *zp2.5* (males only) from 18.1 ng E2/l. This suggests that, compared with vitellogenin, zona pellucida protein and *esr1* transcription is particularly sensitive to estrogen exposure in brown trout, as has been previously reported in other species (18, 30, 54). This corresponds with previous reports that, in the liver, *esr1* is the most responsive estrogen receptor to estrogen exposure and is associated with vitellogeneis (13, 30). There are reports that *esr2a* and/or *esr2b* have a major role in regulating vitellogenesis (33, 52), but our results showed no significant difference in the expression of either of these transcripts or any apparent trends toward this, suggesting that within our experiment, the potential involvement of these genes in vitellogenesis was not regulated by E2-induced changes at the transcriptional level.

#### Novel estrogen-responsive transcripts.

The second most significantly upregulated transcript in fish exposed to 34.4 ng E2/l encoded *nots*. This is a liver-specific aspartic proteinase normally exclusively expressed in the livers of females and has been linked to the proteolytic cleavage of the vitellogenin precursor (44). Although there are several reports of an increase in nothepsin expression following estrogen exposure in fish (44, 60), it has not been widely considered as a particularly estrogen-responsive gene. The degree of induction observed with RNA-Seq in all fish treated with 34.4 ng E2/l and confirmed in all individuals by RT-QPCR (36,000 fold increase for males) is extraordinary and is in the same order of magnitude as the increase in *vtg1* expression. This suggests that nothepsin could serve as a useful indicator of estrogen exposure. However, similar to *vtg1*, there was no increase in *nots* expression quantified by RT-QPCR or RNA-Seq in the lower treatment groups, suggesting nothepsin may be less sensitive to lower estrogen exposure concentrations compared with *esr1* and zona pellucida proteins, at least following this short-term 4-day exposure.

The most downregulated transcript (by 186-fold) in fish exposed to 34.4 ng/l encoded transglutaminase 2-like (*tgm2l*). This strong decrease in expression was confirmed by RT-QPCR (calculated as 250-fold), and *tgm2l* was also found to be significantly downregulated in mature males exposed to 18.1 ng/l. Transglutaminases are a family of enzymes responsible for a diverse range of posttranslational protein modifications by catalyzing the formation of isopeptide bonds (17). *tgm2l* has only been characterized in fish species, and its specific function is not well defined. Here, the striking expression of hepatic *tgm2l* exclusively in sexually mature male trout suggests a role of this gene in male reproductive function. It is possible that, for example, this role may be similar to that of mammalian prostate transglutaminase *tgm4*, which is important in the formation and function of seminal fluid and subsequently influences male fertility (9), but this gene has not been characterized in the majority of fish species. Furthermore, human transglutaminases *tgm2* and *tgm4* are known to have upstream androgen regulatory elements and to be regulated by androgen treatment (12, 25), while gonadal *tgm2* was also upregulated following androgen exposure in juvenile female rainbow trout (3). Recently, we also found reduced expression of *tgm2l* in mature male brown trout exposed to the antiandrogen, linuron (57). Together, this suggests that *tgm2l* may be regulated by androgens and therefore susceptible to disruption by estrogens, which disrupt the androgen-to-estrogen ratio, highlighting a potentially important endocrine biomarker.

#### Estrogen-regulated hepatic processes.

A number of signaling pathways and processes enriched in the list of differentially regulated transcripts can be broadly related to vitellogenesis. Functional analysis revealed enrichment of lipid transport, and also differential regulation of many other transcripts involved in lipid, fatty acid, and cholesterol metabolism. These processes have been previously associated with vitellogenesis in females and E2 exposure in male fish (34, 60) and are likely to reflect the incorporation of lipids into vitellogenins as they are synthesized in the liver.

Additionally, we found evidence of altered regulation of cellular signaling pathways involved in the regulation of hepatic cellular growth and proliferation, in particular IGF signaling and myelocytomatosis oncogene (MYC) signaling. Previously, vitellogenesis in maturing females and estrogen-exposed males has been extensively linked with cellular growth and proliferation in the liver. Transcripts encoding *igfbp* types 2 and 5 were upregulated, while those encoding *igfbp* type 1 were downregulated in fish exposed to 34.4 ng E2/l, suggesting regulation of the transport and bioavailability of IGF1 to bind to its receptors at target cells (24). Cross talk between IGF and estrogen signaling pathways has also been previously demonstrated, and the transcription of *igfbps* is known to be directly regulated by E2 (20, 29). MYC signaling has been proposed as the dominant regulator of estrogen-induced cellular growth, and estrogen exposure induces *myc* transcription via upstream enhancer activation (39). We observed an upregulation of *myc* by up to 10-fold following exposure to 34.4 ng E2/l. Additionally, pathway analysis highlighted its role as a central regulator, alongside *esr1*, of other differentially expressed genes involved in cell proliferation. Tissue homeostasis depends on a balance between cell death and cell survival, growth and proliferation, which are often controlled by the same interacting signaling pathways, including regulation by MYC and IGFs (41). In parallel, apoptosis was among the downregulated cellular processes, suggesting that E2 exposure induced liver growth and proliferation and suppressed apoptosis.

Differential regulation of processes and transcripts involved in methionine and cysteine metabolism were also observed. This pathway plays an important role in regulating DNA methylation, whereby S-adenosylmethionine (SAM) acts as the key methyl group donor. Modulation of DNA methylation has been implicated in cell proliferation and tumorigenesis and reported to be altered by estrogen exposure (2, 37). Additionally, studies in human cell lines have shown that reactive estrogen metabolites (quinones) bind homocysteine, which is a key intermediate in methionine and cysteine metabolism (14). Plasma concentrations of free homocysteine are also regulated by estrogen and are lower in women of reproductive age (11). Therefore, a reduction in homocysteine might contribute to the observed differential regulation of these associated metabolic enzymes.

Exposure to E2 also resulted in upregulation of a number of transcripts with roles in transcription and translation, as well as an overrepresentation processes and pathways involved in their regulation. Furthermore, ribosome and endoplasmic reticulum were among the most enriched GO and KEGG pathway terms. Ribosome biogenesis in response to estrogen exposure has been previously linked to increased cell growth and proliferation, reflecting a general upregulation of translation (39, 60). In fish, the observed induction of transcription and translation machinery is also likely to reflect the very significant increase in the synthesis and posttranslational modification of vitellogenins and zona pellucida proteins. Ribosomal constituent overexpression has been previously reported in male zebrafish exposed to E2 (47) and in female vitellogenic livers (60). Together, induction of the expression of growth regulators, and of transcription and translation pathways, illustrates the very significant stimulatory effect of E2 on cell proliferation and protein synthesis in the livers of male brown trout.

A number of processes involved in carbohydrate and amino acid metabolism dominate the overrepresented GO terms in the list of downregulated transcripts. In particular, there was a striking downregulation of transcripts associated with gluconeogenesis, including tyrosine, alanine, aspartate, and glutamate metabolic pathways. Transcripts encoding a key gluconeogenic enzyme, tyrosine aminotransferase (*tat*), were amongst the most significantly downregulated transcripts in the 34.4 ng E2/l-exposed fish, and this was also confirmed by RT-QPCR analysis. Additionally, a large number of other transcripts involved in gluconeogenesis were also downregulated, including a transcript encoding the rate limiting enzyme phosphoenolpyruvate carboxylate 1 (*pck1*). A decrease in liver glucose concentration has previously been reported in vitellogenic female fish, as well as immature trout treated with estrogen and has been shown to be primarily associated with reduced gluconeogenesis rather than increased glucose utilization (59). In mammalian studies, estrogen signaling has been widely shown to regulate energy metabolism, including through both glucose and lipid metabolic pathways (8, 22, 46). In the present dataset, there was also evidence of upregulation of lipid synthesis and transport, possibly suggesting a shift toward lipid metabolism as a preferable energy source for vitellogenesis.

#### Application of RNA-Seq in ecotoxicology.

Together with a number of previous studies [see review by Qian et al. (42)], the present study provides evidence that RNA-Seq has very significant potential for mechanistic analysis of chemical exposures in (non-) model organisms and also offers a number of technical advantages over other global methodologies to measure global transcript expression. Here, we successfully identified highly conserved responses to estrogen, compared with other species, together with several more novel estrogen-responsive transcripts. This highlights the potential for RNA-Seq to investigate mechanisms of toxicity for less-studied chemical pollutants and also demonstrates the feasibility of conducting global gene expression profiling in species of environmental interest for which previous sequence information is limited, without the investment required to develop a specific microarray or the need to use surrogate model species.

Analysis of spike-in controls provided strong technical validation for the accuracy of the expression analysis, and the mean calculated dynamic range in expression measured in our experimental data based on the quantified expression of control transcripts was 26,753, which far exceeds that typically found in microarray experiments (up to several hundred fold)(6). The very small number of differentially expressed transcripts between control treatments and also between the control group and groups exposed to 1.9 and 18.1 ng E2/l reflects the ability of the technique to avoid false positives. This is consistent with other studies that have reported that RNA-Seq is accurate and reproducible. However, there is also some evidence that the stringent statistical thresholds imposed during RNA-Seq analysis can reduce its relative sensitivity, particularly for rare transcripts or those with small fold-changes between treatment groups (36, 43, 49). Here, we found evidence that RNA-Seq was less sensitive than RT-QPCR (using more individual fish per treatment) for detecting significant changes in expression of transcripts in the lower concentration groups, although clear trends toward differential regulation were apparent in some cases in the RNA-Seq dataset. It is important to note that we used only three individual fish per treatment group for the RNA-Seq analysis, and this is likely to have contributed to the low sensitivity of the technique in this study.

Using individual fish within an RNA-Seq experimental design provides a considerable advantage for statistical power compared with the use of pooled replicates, and maximizing the number of biological replicates is essential to reduce the impact of biological variation between individuals and likely to increase sensitivity for detection of transcripts with small fold-changes in expression. In the present study we analyzed three individual fish per treatment group, and we would expect sensitivity to improve considerably with the use of more replicates. Maximizing the sequence coverage depth per sample is also likely to increase the sensitivity of RNA-Seq, particularly for rare transcripts, and is therefore equally important to consider. The feasibility of maximizing both the number of replicates and coverage in RNA-Seq experiments is rapidly improving with developments in sequencing technology and is likely to considerably improve sensitivity in the future. Overall, our data highlight the potential of RNA-Seq as a valuable tool in mechanistic ecotoxicology that, crucially, is not reliant on pre-existing genomic resources for the species of interest.

## GRANTS

This work was supported by a Natural Environment Research Council CASE PhD studentship (Grant NE/I528326/1) and the Salmon & Trout Association. Karen Moore was supported by a Wellcome Trust Institutional Strategic Support Award (WT097835MF).

## DISCLOSURES

No conflicts of interest, financial or otherwise, are declared by the author(s).

## AUTHOR CONTRIBUTIONS

Author contributions: T.M.U.W. and E.M.S. conception and design of research; T.M.U.W. and E.M.S. interpreted results of experiments; T.M.U.W. and E.M.S. edited and revised manuscript; T.M.U.W., J.A.S., K.M., and E.M.S. approved final version of manuscript; T.M.U.W., J.A.S., K.M., and E.M.S. performed experiments; T.M.U.W. analyzed data; T.M.U.W. prepared figures; T.M.U.W. drafted manuscript.

## Supplementary Material

Table S1

## Footnotes

1 The online version of this article contains supplemental material.
