# Supplementary material for: Identification of conserved hepatic transcriptomic responses to 17β-estradiol using high-throughput sequencing in brown trout
Source: Physiol Genomics. 2015 Jun 16;47(9):420–31. doi: 10.1152/physiolgenomics.00123.2014 (PMC4556936; doi:10.1152/physiolgenomics.00123.2014)
Supplement: Table S1 [file Table_S1.pdf]

Supplemental Table: Putative cumulus or granulosa cell biomarkers correlated to oocyte quality and developmental competence

Gene Symbol	Description
PSMA2	proteasome (prosome, macropain) subunit, alpha type, 2
TMEM126A	transmembrane protein 126A
TMEM126B	transmembrane protein 126B
PSMA2	proteasome (prosome, macropain) subunit, alpha type, 2
PSMA3	proteasome (prosome, macropain) subunit, alpha type, 3
LOC100295249	similar to homeobox C8
PSMA2	proteasome (prosome, macropain) subunit, alpha type, 2
ATP5J	ATP synthase, H+ transporting, mitochondrial F0 complex, subunit F6
DIAPH3	diaphanous homolog 3 (Drosophila)
C4H7ORF11	chromosome 7 open reading frame 11 ortholog
COX7C	cytochrome c oxidase subunit VIIc
VCAN	versican
LOC100295268	hypothetical protein LOC100295268
DBI	diazepam binding inhibitor (GABA receptor modulator, acyl-Coenzyme A binding protein)
PSMA5	proteasome (prosome, macropain) subunit, alpha type, 5
WDR89	WD repeat domain 89
COPS5	COP9 constitutive photomorphogenic homolog subunit 5 (Arabidopsis)
RSRC1	arginine/serine-rich coiled-coil 1
CNO	cappuccino homolog (mouse)
APCDD1	adenomatosis polyposis coli down-regulated 1
TOMM5	translocase of outer mitochondrial membrane 5 homolog (yeast)
PLCB4	phospholipase C, beta 4
LOC509351	hypothetical LOC509351
FAM92A1	family with sequence similarity 92, member A1
NOL7	nucleolar protein 7, 27kDa
PSMD14	proteasome (prosome, macropain) 26S subunit, non-ATPase, 14
ACYP1	acylphosphatase 1, erythrocyte (common) type
SEMA6D	sema domain, transmembrane domain (TM), and cytoplasmic domain, (semaphorin) 6D
DBI	diazepam binding inhibitor (GABA receptor modulator, acyl-Coenzyme A binding protein)
TCEB1	transcription elongation factor B (SIII), polypeptide 1 (15kDa, elongin C)
LOC100295873	similar to cytochrome c oxidase, subunit VIIc
NOL7	nucleolar protein 7, 27kDa
NUDCD2	NudC domain containing 2
GTF3C6	general transcription factor IIIC, polypeptide 6, alpha 35kDa
APCDD1	adenomatosis polyposis coli down-regulated 1
LOC100336216	cytochrome c oxidase, subunit VIIc-like
LOC100297413	similar to cytochrome c oxidase, subunit VIIc

RNASEH2B	ribonuclease H2, subunit B
LDLR	low density lipoprotein receptor
SRXN1	sulfiredoxin 1 homolog (S. cerevisiae)
SCG2	secretogranin II (chromogranin C)
CTSS	cathepsin S
VNN1	vanin 1
MXI1	MAX interactor 1
DUSP1	dual specificity phosphatase 1
ADORA2B	adenosine A2b receptor
SHISA2	shisa homolog 2 (Xenopus laevis)
KLF4	Kruppel-like factor 4 (gut)
N4BP2L1	NEDD4 binding protein 2-like 1
PCBD1	pterin-4 alpha-carbinolamine dehydratase/dimerization cofactor of hepatocyte nuclear factor 1 alpha
BOLA-DQB	major histocompatibility complex, class II, DQ beta
YTHDF1	YTH domain family, member 1
AMY2A	amylase, alpha 2A (pancreatic)
TMEM50B	transmembrane protein 50B
IER3	immediate early response 3
ZFP36L1	zinc finger protein 36, C3H type-like 1
MYLIP	myosin regulatory light chain interacting protein
BHLHB2	basic helix-loop-helix domain containing, class B, 2
PLAT	plasminogen activator, tissue
LITAF	lipopolysaccharide-induced TNF factor
ETS2	v-ets erythroblastosis virus E26 oncogene homolog 2 (avian)
SIPA1L2	signal-induced proliferation-associated 1 like 2
SIPA1L2	signal-induced proliferation-associated 1 like 2
SYTL2	synaptotagmin-like 2
NCKAP5L	NCK-associated protein 5-like
HPCAL1	hippocalcin-like 1
COL4A1	collagen, type IV, alpha 1
ADAMTS1	ADAM metallopeptidase with thrombospondin type 1 motif, 1
RBM39	RNA binding motif protein 39
GOLGA7B	golgi autoantigen, golgin subfamily a, 7B
DAB2	disabled homolog 2, mitogen-responsive phosphoprotein (Drosophila)
RBM39	RNA binding motif protein 39
LOC100295764	similar to WD repeat and SOCS box-containing 1
ARHGEF3	Rho guanine nucleotide exchange factor (GEF) 3
FOS	FBJ murine osteosarcoma viral oncogene homolog
ELL2	elongation factor, RNA polymerase II, 2
TLE1	transducin-like enhancer of split 1 (E(sp1) homolog, Drosophila)
C27H8orf4	chromosome 8 open reading frame 4 ortholog
GRASP	GRP1 (general receptor for phosphoinositides 1)-associated scaffold protein
EGR1	early growth response 1

HERPUD1	homocysteine-inducible, endoplasmic reticulum stress-inducible, ubiquitin-like domain member 1
